# Capping Actin Protein Overexpression in Human Colorectal Carcinoma and Its Contributed Tumor Migration

**DOI:** 10.1155/2018/8623937

**Published:** 2018-08-01

**Authors:** Tsung-Jung Tsai, Yun-Ping Lim, Wen-Ying Chao, Chien-Chin Chen, Yi-Ju Chen, Ching-Yen Lin, Ying-Ray Lee

**Affiliations:** ^1^Department of Gastroenterology, Ditmanson Medical Foundation Chiayi Christian Hospital, Chiayi, Taiwan; ^2^Department of Pharmacy, College of Pharmacy, China Medical University, Taichung 404, Taiwan; ^3^Department of Nursing, Min-Hwei College of Health Care Management, Tainan 736, Taiwan; ^4^Department of Pathology, Ditmanson Medical Foundation Chiayi Christian Hospital, Chiayi, Taiwan; ^5^Department of Cosmetic Science, Chia Nan University of Pharmacy and Science, Tainan, Taiwan; ^6^Department of Medical Research, Ditmanson Medical Foundation Chiayi Christian Hospital, Chiayi, Taiwan

## Abstract

**Objective:**

Human colorectal cancer (CRC) is the third most common cancer; patients with metastatic colorectal cancer (mCRC) show poor prognosis than those with CRC cases. There are no reliable molecular biomarkers for the diagnosis of CRC prognosis except with pathological features. Therefore, it is urgent to develop a biomarker for diagnosis and/or prediction of human CRC. In addition, capping actin protein (CapG) belongs to the gelsolin family and has been reported to contribute on tumor invasion/metastasis in multiple human cancers. Here, we are the first to evaluate the expression of CapG in human CRCs.

**Study Design:**

To investigate the expression levels of CapG in human tissue array by immunohistochemistry (IHC) staining. Moreover, the mRNA and protein levels were also confirmed in four CRC cell lines and determined using real-time RT-PCR and Western blotting. Finally, a Matrigel transwell invasion assay was used to evaluate the invasion ability in CapG high or low expression cells.

**Results:**

We demonstrated that CapG could be determined in the normal colon tissue and human CRC specimens. However, CapG was significantly overexpressed in the mCRC specimens compared with that in CRC specimens and normal cases. It was also detectable in the four CRC cell lines including mRNA and protein levels. We also found that knockdown of the expression of CapG reduced tumor migration.

**Conclusions:**

In this study, we suggested that CapG could be used as a biomarker for metastatic CRC in the clinical specimens. Moreover, our *in vitro* study demonstrated that CapG might contribute on tumor metastasis in human CRCs.

## 1. Introduction

Colorectal cancer (CRC) is the third most common cancer and the fourth most common cause of cancer-related death in men and women worldwide. The risk factors for CRC are age, lifestyle, familial history of CRC, and patients with inflammatory bowel disease (IBD) and Crohn's disease [1]. Current clinical treatment strategy for CRC includes surgical resection, chemotherapy, and target therapies. However, the prognosis of patients with CRC is determined by pathological features and the diagnostic stage of the tumor. In patients with in situ carcinoma of CRC, the 5-year survival rate is 90%. However, patients with metastatic colorectal cancer (mCRC) have poor prognosis, and the five-year survival rate is 10–20% [2]. The target therapies including monoclonal antibodies against receptor tyrosine kinases (RTK) such as epidermal growth factor receptor (EGFR) may contribute to the improvement in survival of mCRC patients. Therefore, early diagnosis for CRC patients or identification of predictive markers for treatment of mCRC patients is urgent.

The main cause of death from CRC is usually related to metastasis and resistance to chemotherapeutics after surgery. If prognosis could be determined more precisely before treatment, patients would be more effectively treated with individualized therapy. The spread of solid tumor from the primary site and subsequent dissemination is facilitated by detachment of malignant cells. This metastatic cascade is critically contributed by the cytoskeleton and extracellular matrix (ECM) remodeling through migratory and invasive signal regulation [3, 4].

CapG, a membrane of the gelsolin family, has the common property of binding to the barbed end of actin filaments with high affinity and has been identified as playing important roles in tumor invasion and metastasis including breast, lung, and prostate carcinoma [5–7]. CapG is contributing on the modulation of cellular motility through interacting differentially with the actin cytoskeleton [8]. It also has been reported to act on the tumor invasion and migration [5, 6, 8]. Although gelsolin has been reported to overexpress and induce tumor invasion in CRC cases [6], the role and the association of CapG with CRC are still unclear.

In this study, we identified the expression of CapG from immunohistochemical staining on tissue microarray (TMA) of CRC patients and carried out clinical validation with pathology. Moreover, the mRNA and protein levels of CapG are also evaluated in the various CRC cell lines. Finally, cellular invasion is also confirmed in the CapG-overexpressed cell lines.

## 2. Materials and Methods

### 2.1. Patient Samples

A human colorectal cancer-metastasis-normal tissue microarray slide (CDA3) was purchased from SuperBioChips Laboratories (Seoul, Korea). A total of 50 patients (including 10 patients with metastatic carcinoma) who were pathologically diagnosed with CRC and 9 normal cases were selected and included in this TMA (the data sheet: http://www.tissue-array.com/product/product_view.php?product_code=22&category1=1&category2=3&category3=14). However, in this slide, there were two cases exceptionally having no tumor cells. The diagnosis with tumor stage and pTNM in these cases could be found in the TMA data sheet. Moreover, the prognosis of these cases also could be found in the supplementary data of this TMA. This study was approved by the Institutional Review Board (IRB) of Ditmanson Medical Foundation Chiayi Christian Hospital (IRB106018).

### 2.2. Cell Lines and Cell Culture

Human colorectal cancer cell lines, including HCT116, HT29, SW1116, and DLD-1, were obtained from ATCC. HT29 and DLD-1 cells were cultured in Dulbecco's Modified Eagle's Medium (DMEM); HCT116 cells were cultured in RPMI 1640 medium, and SW1116 cells were cultured in L-15 medium (all media were purchased from Gibco BRL, Grand Island, NY). All media were supplemented with 10% fetal bovine serum (FBS) (Trace Biosciences, Sydney, Australia), penicillin (200 U/ml; Biowest, Nuaillé, France), and streptomycin (100 *μ*g/ml; Bioweat). Cells were maintained at 37°C with 5% CO_2_.

### 2.3. Immunohistochemistry Staining

Tissue specimens in the TMA were deparaffinized, boiled in citric acid, and treated with hydrogen peroxide before incubation with anti-CapG primary antibody (GeneTex Inc., Hsinchu, Taiwan) overnight at 4°C. Then, the TMA was incubated with polymer-horseradish peroxidase-conjugated secondary antibody at room temperature. Specimens were developed with diaminobenzidine (DAB) and counterstained with hematoxylin (Dako, Glostrup, Denmark). The images were determined with an Olympus microscope. The intensity of staining was graded from 0 (undetectable), 1 (weak staining), 3 (medium staining) to 5 (intense staining), while the proportion of positive staining tumor cells within a tissue was scored from 0% to 100% of tumor cells identified. The staining score was expressed as the product of intensity of staining and proportion of tumor positivity.

### 2.4. Real-Time Quantitative RT-PCR

Real-time PCR was used to evaluate the expression of CapG in the four CRC cell lines. The total RNAs from HCT116, HT29, SW1116, and DLD-1 cells were extracted by RNA isolation kit (GE Healthcare, Munich, Germany), and the cDNAs were prepared by using Transcriptor First Strand cDNA Synthesis Kit (Roche, Mannheim, Germany), carried out on the CFX96 Real-Time System (Bio-Rad). The conditions of real-time PCR were as follows: one cycle at 50°C for 2 min and 95°C for 10 min and followed by 40 cycles of denaturation at 95°C for 15 seconds and annealing extension at 55°C for 1 min. The primers used included the following:

1. CapG-F: CGAACACTCAGGTGGAGATT
2. CapG-R: TCCAGTCCTTGAAAAATTGC
3. GAPDH-F: TGCACCACCAACTGCTTAGC
4. GAPDH-R: GGCATGGACTGTGGTCATGAG

### 2.5. Western Blotting

Total cell lysates of human colorectal cancer cell lines, HCT116, HT29, SW1116, and DLD-1, were extracted with RIPA buffer (containing PMSF, EGTA, aprotinin, leupeptin, Na_3_VO_4_ (Sigma, MO, USA), and EDTA (Merck, Darmstadt, Germany)). Proteins from each sample were analyzed with 10% sodium dodecyl sulfate polyacrylamide gel electrophoresis (SDS-PAGE), transferred to PVDF membrane (Bio-Rad Laboratories Inc., CA, USA), and blocked and incubated with primary antibody (CapG antibody; GeneTex Inc.) overnight at 4°C followed by secondary antibody staining for 1 h at room temperature. Finally, the data were examined using the BioSpectrum Imaging system (UVP, CA, USA). The expression levels of the proteins in the cells were quantified using Image-J software.

### 2.6. Migration Assay

A control shRNA (CCGGACACTCGAGCACTTTTTG) and CapG shRNA (CCGGCCGAACACTCAGGTGGAGATTCTCGAGAATCTCCACCTGAGTGTTCGGTTTTT) were transfected into HT29 cells, and the migration activity of the cells was determined under transwell migration assay. 1 × 10^5^ cells/well were seeded and cultured in the transwell upper chamber for 24 h, and 10% FBS was then used as chemoattractant in the bottom chamber for 24 h. Cells migrated in the bottom chamber were stained with 2% crystal violet solution (Sigma, MO, USA).

### 2.7. Statistical Analysis

All data were analyzed using GraphPad Prism for Windows, version 6 (GraphPad Software Inc., San Diego, CA, USA). The data presented as the mean ± SD, and *p* values was calculated by Microsoft Excel and SPSS, version 21.0 (IBM SPSS Statistics, USA) through the nonparametric tests. *p* values less than 0.05 were considered as “statistically significant”.

## 3. Results

### 3.1. CapG Expression Is Illustrated in the Human Metastatic Colorectal Carcinoma

**Figure 1:**
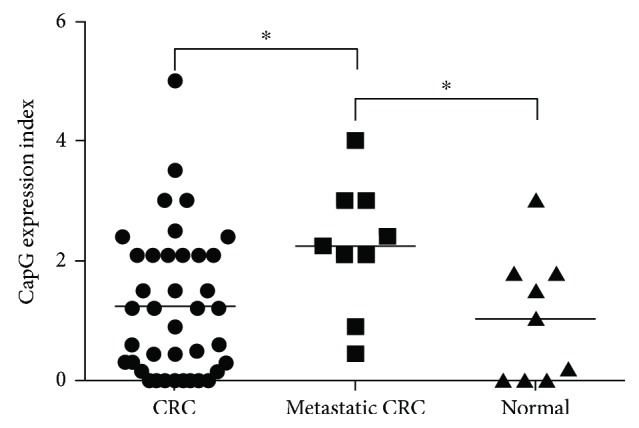
CapG expresses in the human colorectal carcinoma and normal specimens in tissue microarray. A tissue microarray was used to examine the expression of CapG by immunohistochemistry; the expression index of CapG in the human colon tissues was quantified by a pathologist, and scores of the specimens were also organized depending on the pathologic diagnosis with normal, colorectal carcinoma, and metastatic colorectal carcinoma. ^∗^ *p* < 0.05.

The demographic data and the clinical characteristics of the patients were showed in Table 1. The expression index of CapG in these specimens was definite into high (expression index ≧ 1.5) and low (expression index < 1.5). There were no significant difference regarding CapG expression between groups including age, sex, tumor differentiation, tumor size and invasive, lymph node status, and tumor stage (Table 1). The expression of CapG in the CRC clinical specimens including 9 normal cases, 39 CRC cases, and 9 metastatic CRC cases was validated by IHC staining and determined under microscopy (Supplementary data 1). CapG expression in tumor tissues as well as in normal tissues was determined by Dr. Chen Chien-Chin (a clinical doctor of pathology) and scored for the intensity of staining (nonexpression: 0, weak expression: 1, medium expression: 3, and intense expression: 5) and proportion of tumor positivity (0–100%). CapG overexpression was significantly determined in the metastatic CRC specimens compared with that in CRC specimens and normal cases (Figure 1). This finding was consistent with the reports in various human cancers [5, 6]. However, our data also showed that no statistically significant expression of CapG between CRC cases and normal specimens (Figure 1). Herein, our data suggested that CapG might be a prognostic marker for metastasis but could not be used as a diagnostic tumor marker for CRC patients.

### 3.2. mRNA and Protein Expression Levels of CapG in the Human Colorectal Carcinoma Cell Lines

**Figure 2:**
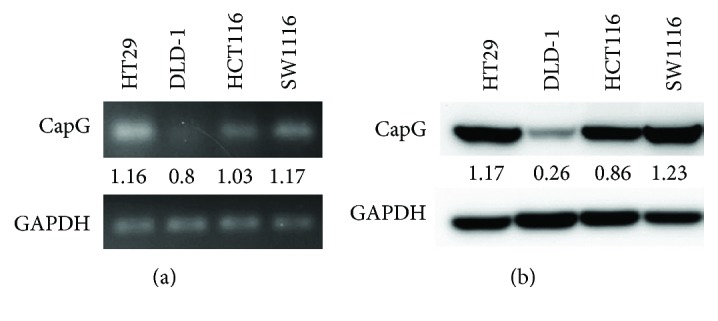
The mRNA and protein expression levels of CapG in four human colorectal carcinoma cell lines. Four human CRC cell lines including HT29, DLD-1, HCT116, and SW1116 were used to analyze the expressions of (a) mRNA and (b) protein of CapG. GAPDH was used as a loading control.

In order to evaluate the expressions and the further bioactivity of CapG in the human CRC cells, four CRC cell lines were examined. Human CRC cell lines including SW1116, HT29, HCT116, and DLD-1 were cultured, and the mRNA and total protein of CapG were determined by real-time RT-PCR and Western blotting.  Figure 2(a) showed that the expression level of CapG mRNA in these cells was SW1116 > HT29 > HCT116 > DLD-1. Consistently, the protein expression level of CapG in these cells was SW1116 > HT29 > HCT116 > DLD-1 (Figure 2(b)). Here, we firstly illuminated the differential expressions of mRNA and protein of CapG in SW1116, HT29, HCT116, and DLD-1 cells.

### 3.3. Knockdown of the Expression of CapG in HT29 Cells Reduces the Cellular Migration Ability

**Figure 3:**
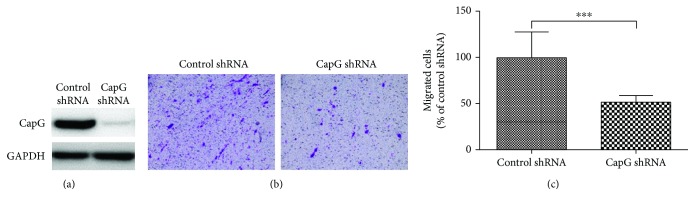
The ability of cellular migration was determined in HT29 cells. (a) A control shRNA and a CapG shRNA were transfected separately into HT29 cells, and the protein expression of CapG was determined by Western blotting. (b) A transwell migration assay was used to evaluate tumor migration ability in cells after 24 h chemoattractant with FBS. Invaded cells in the bottom chamber were stained with 2% crystal violet solution, and (c) the numbers were counted. ^∗∗∗^ *p* < 0.001.

Our data (Figure 1) illustrates that CapG was overexpressed in the metastatic CRC cases, suggesting that CapG may contribute on tumor metastasis in human CRC. To address this hypothesis, a shRNA of CapG was transfected into HT29 cells and the cellular migration was further determined under a transwell migration assay.  Figure 3(a) demonstrated that CapG shRNA could significantly suppress the expression of CapG in HT29 cells. Moreover, the migration ability was reduced in HT29 cells with CapG knockdown (Figures 3(b) and 3(c)). These findings suggested that CapG overexpression in the human colorectal cancer cells might contribute on the tumor cell migration.

## 4. Discussion

In this study, we are the first to address the expression of CapG in human colorectal cancers. IHC staining showed that CapG was highly expressed in metastatic CRC specimens compared with that in CRC specimens and normal cases (Figure 1). It indicated that high expression of CapG might be correlated with the migration of CRC, which might be a useful prognostic marker for the early diagnosis of mCRC. Importantly, our finding was consistent with previous findings in human glioma [9], breast cancer [10], gastric cancer [11], and ovarian cancer [12], suggesting that CapG could be an oncoprotein. In addition, the previous report showed that patients with lymph node metastases were associated with overexpression of CapG in 75 pulmonary adenocarcinomas [13]. Among them, overexpression of CapG in the advanced stage of pulmonary adenocarcinoma (stages III and IV) was also higher than that in the earlier stages of pulmonary adenocarcinomas [13]. Most of the literatures have demonstrated that CapG is associated with invasion and migration of cancer cells. The previous study demonstrated CapG lost exhibiting in the small-cell lung cancer (H69, Lu22, Lu139, Lu134, and H209), lung adenocarcinoma (PC7, RERF-LCMS), gastric cancer (AZ521), and melanoma (A2058) [14]. To test ectopic CapG from the tumorigenic stages of human diploid fibroblast strain (RBT) and gastric cancer cell line AZ521, the results showed that CapG overexpression could suppress tumorigenicity but showed no influence in the anchorage-independent growth of RBT and AZ521 cells and might be a candidate tumor suppressor [14]. CapG protein is known to possess actin-modulating activity [15], and it is possible that an alteration of microfilaments contributes to the acquisition or loss of tumorigenicity.

Furthermore, CapG is belonging in the gelsolin/villin family of actin-regulatory proteins. Gelsolin is associated with the invasion and metastasis of cancer cells, such as prostate cancer [7] and lung adenocarcinoma [13]. Gelsolin promotes invasion and metastasis of HCT116 and DLD-1 cells via modulation of the invasion-associated urokinase-type plasminogen activator (uPA) [6]. Moreover, the uPA system plays the role in causing aggressive tumor behavior, which promotes invasion and metastasis in several tumors [6]. Westbrook et al. demonstrated that CAPG and PDZ domain-containing protein GIPC1 (GIPC1) were independently associated with bone metastasis for breast cancer [10]. CAPG showed a weak association, and GIPC1 expressed a stronger relation with bone metastasis [10]. Moreover, CRCs are classified as microsatellite instability (MSI) or microsatellite stable (MSS). To further confirm the role of CapG in human CRC, shRNA of CapG was transfected into HT29 cells and the inhibition of cellular migration was also demonstrated (Figure 3). Therefore, we suggest that CapG may play the role of tumor metastasis in human colorectal cancers.

## 5. Conclusion

CapG has been showed to overexpress and contribute on malignancy in multiple human cancers. However, the expression and the role of CapG in the human colorectal cancers are still unknown. In the present study, we demonstrated that CapG could be determined in the normal colon tissue and human CRC specimens. Most importantly, CapG was significantly overexpressed in the metastatic CRC specimens compared with that in CRC specimens and normal cases. Further study demonstrates that knockdown of the expression of CapG in human CRC cells decreases the tumor migration ability. Our in vitro study suggests that CapG could be used as a biomarker for metastatic CRC in the clinical specimens and might play a role in tumor metastasis.

## Figures and Tables

**Table 1:** Distribution of characteristics of patients and the expression index of CapG.

CapG immunostaining intensity				
Clinical classification	Total number	Low (number)	High (number)	*p* value
Gender				*p* = 0.988 (male versus female)
Male	39	20	19	
Female	18	10	8	
Age (years)				*p* = 0.789 (≥55 versus <55)
≥55	30	14	16	
<55	26	15	11	
T-primary tumor				*p* = 0.742 (T1 + T2 versus T3 + T4)
T1 + T2	3	2	1	
T3 + T4	36	22	14	
Lymph node status				*p* = 0.902 (negative versus positive)
Negative	14	8	6	
Positive	25	15	10	
Stage				*p* = 0.399 (I + Π versus III + IV)
I + Π	13	7	6	
III + IV	26	16	10	
Differentiation				*p* = 0.827 (pairwise comparison randomly)
Well differentiation	10	5	5	
Moderate differentiation	21	13	8	
Poor differentiation	3	2	1	

## Data Availability

The data used to support the findings of this study are available from the corresponding author upon request.

## Supplementary Materials

Supplementary Materials Supplementary data 1: CapG expresses in the human colorectal carcinoma and normal specimens in tissue microarray. The tissue microarray was used to examine the expression of CapG by immunohistochemistry, and the photographs were determined by microscopy. There were no tumor specimens in the samples number 35 and number 45. A blank was showed in the number 60.
