# Supplementary figures and images for: Capping Actin Protein Overexpression in Human Colorectal Carcinoma and Its Contributed Tumor Migration
Source: Anal Cell Pathol (Amst). 2018 Aug 1;2018:8623937. doi: 10.1155/2018/8623937 (PMC6093051; doi:10.1155/2018/8623937)

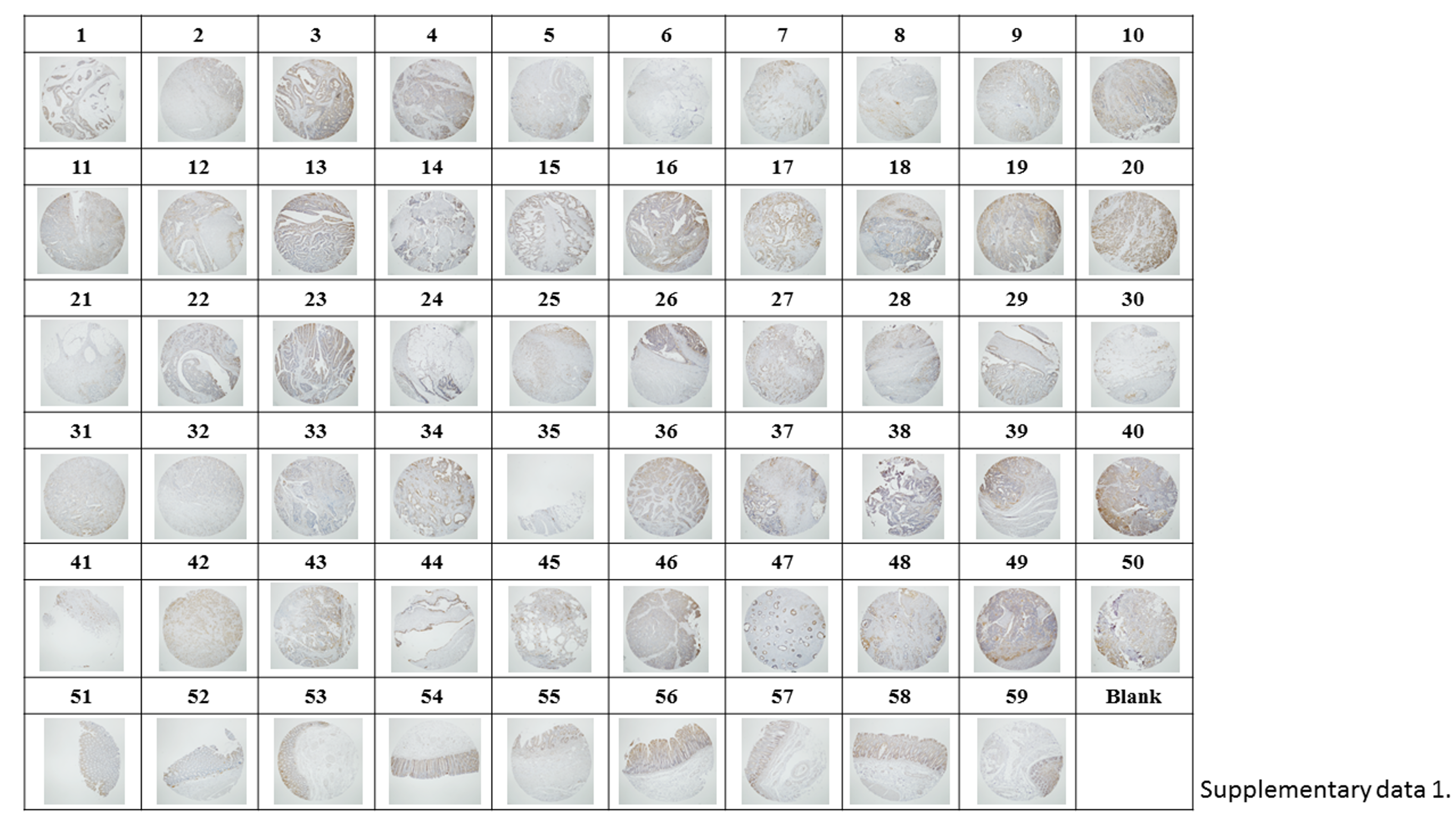

Supplement: Supplementary Materials — Supplementary data 1: CapG expresses in the human colorectal carcinoma and normal specimens in tissue microarray. The tissue microarray was used to examine the expression of CapG by immunohistochemistry, and the photographs were determined by microscopy. There were no tumor specimens in the samples number 35 and number 45. A blank was showed in the number 60. [file 8623937.f1.tif]
